# Comparative study between high and low dose methylene blue infusion in septic cancer patients: a randomized, blinded, controlled study

**DOI:** 10.1186/s12871-024-02792-3

**Published:** 2025-01-08

**Authors:** Ehab Hanafy Shaker, Ahmed Mohamed Soliman, Ahmed Abd Elmohsen Bedewy, Mai Mohamed Elrawas

**Affiliations:** 1 Department of Anaesthesia, Intensive care and Pain management, National Cancer Institute, Cairo University, Cairo, Egypt; 2 Department of Anaesthesia and Surgical Intensive Care, Faculty of Medicine, Helwan University, Cairo, Egypt

**Keywords:** Septic shock, Methylene blue, Cancer, Noradrenaline

## Abstract

**Purpose:**

Septic shock is a common threat, and is the primary cause of death in almost all critical care units. Mortality of septic shock remains exceedingly high. The early use of methylene blue (MB) in different doses as adjunctive to vasopressors has promising results.

**Methods:**

This double-blind, randomized, controlled trial comprised 90 patients divided into 3 groups: Group A received a 100 ml 0.9% NaCl placebo over 20 min; Group B received an MB bolus of 1 mg/kg in 100 ml 0.9% NaCl, and Group C received MB bolus of 4 mg/kg in 100 ml 0.9% NaCl during the same period. Groups B and C were given a 0.25 mg/kg/hour infusion of MB for 72 h after the bolus dose. All patients were started on noradrenaline at an infusion rate of 0.1–0.2 µ/kg/min and were adjusted accordingly to maintain MAP ≥ 65 mmHg. Time of vasopressor discontinuation was the primary outcome while total doses of vasopressors, ventilation days, vasopressors free days, total ICU stay, total hospital stay, and mortality rate were the secondary outcomes.

**Results:**

Groups B and C exhibited significantly decreased time to vasopressor termination, and vasopressor-free days at 28 days in comparison to Group A. However, there was no significant difference between Groups B and C. Groups B and C had significantly lower noradrenaline dosages compared to Group A, however, no significant difference between Group B and Group C was found. The difference between the three groups in mortality rate was near statistical significance (*p* = 0.083). Using the logistic regression model, the 4 mg/kg group was protective against mortality with a hazard ratio of 0.29 (95%CI: 0.09–0.90).

**Conclusion:**

In cancer patients with septic shock, early adjunctive MB delivery reduces the time to a vasopressor stoppage and increases the vasopressor-free days. No significant difference between high and low MB bolus doses, and no significant adverse effects were noted. Compared to placebo, the 4 mg/kg bolus dose shows a survival advantage.

**Trial registration:**

Prospectively registered at clinicaltrials.gov [NCT 06005558]. (Date of registration 15/08/2023).

## Introduction

Sepsis is a potentially fatal organ failure that can arise from an overreaction of the host to an infection [1]. Despite being the leading cause of mortality within hospitals [2], it is treatable with timely and early therapies [3].

The Third International Consensus definitions, or Sepsis-3, are used in the 2021 Surviving Sepsis Campaign (SSC). Sepsis was defined as “life-threatening organ dysfunction caused by a dysregulated host response to infection”. A score of two or more on the Quick Sequential (Sepsis-related) Organ Failure Assessment (qSOFA) indicates organ dysfunction. Septic shock is a subset of sepsis in which major circulatory, cellular, and metabolic changes are associated with higher mortality than with sepsis alone. In euvolemic patients, to be diagnosed with septic shock a patient must be on a vasopressor to reach a mean arterial pressure (MAP) ≥ 65 mmHg and with a serum lactate above 2 mmol/L [1].

The cornerstone of treating septic shock is administering, in the first hour, intravenous fluids, vasopressors, and antibiotics [4]. The goal of management is to keep the patient hemodynamically stable until the antibiotics start working to combat the infection. Low-dose corticosteroids are prescribed for non-responders. Further research is required to determine whether other medications can aid non-responders as well as to enhance overall outcomes. A nitric oxide inhibitor called methylene blue (MB) can reverse the vasodilatation that occurs early in septic shock [5]. Vasodilatation is caused by soluble guanylyl cyclase (sGC), which is activated by nitric oxide (NO). Cyclic guanosine monophosphate (cGMP)-dependent protein kinases (PKGs) are then activated [6]. Methylene blue inhibits inducible nitric oxide synthase (iNOS) and blocks sGC with selectivity. As a result, it affects the microcirculation only slightly. Intravenous MB acts within 30 to 60 min. Peak concentration is at 30 min. In addition to the kidneys, it is eliminated by the bile and fecal pathways. A plasma half-life of 5 to 6 h has led multiple investigators to administer a 0.25-2 mg/kg/h infusion for a duration of up to 3 days after a bolus dosage [7–9].

The aim of the trial is to compare the effects of high and low bolus doses of MB in sepsis in cancer patients. The main hypothesis of the study is that MB infusion would decrease the need for vasopressor infusion and that the initial high bolus dose of MB would be more beneficial than the low dose.

## Patients and methods

After approval of the ethical committee at the National Cancer Institute (NCI) - Cairo University (AP2307-501-058) and registration at clinicaltrials.gov (NCT 06005558), all patients’ guardians gave written informed consent. This randomized controlled trial involved 90 patients with various types of cancers admitted to the intensive care unit of the National Cancer Institute with septic shock in the period from 8/2023 to 4/2024. Data was collected anonymously and all participants’ guardians were informed about the target and benefits of the study.

Patients who met the Sepsis-3 criteria (confirmed infection, needing a vasopressor to maintain a MAP of 65 mmHg and serum lactate > 2 mmol/L following adequate fluid resuscitation), plus requiring mechanical ventilation were included. Those who were at least 18 years old and ≤ 65 years old were eligible for inclusion. A myocardial infarction or cerebrovascular accident within the previous three months, Glucose-6-phosphate dehydrogenase deficiency, a known allergy to MB or food dyes, nitrates use within the previous 72 h, severe liver, kidney, or lung disease (creatinine > 3.5 mg/dL), pregnancy, more than 24 h after the start of noradrenaline, other types of shock including hemorrhagic, obstructive or hypovolemic, recent (4 weeks) intake of selective serotonin reuptake inhibitors or MAOi, and the patient guardian’s refusal were the exclusion criteria.

### Randomization and blinding

The participants were allocated to three treatment groups by a preset randomization sequence that was prepared in sealed opaque envelopes. The sequence was computer-generated using a 1:1 allocation ratio and permuted blocks of size 2. Intervention assignment was the responsibility of the ICU physicians. The study lacked complete blinding due to the presence of bluish coloration on the skin and secretions caused by the administration of methylene blue. However, the allocation to low and high MB doses – which was the second objective of the study - was blinded to the patients and outcome assessors. The study drugs were prepared by an independent pharmacist.

Group A (*n* = 30) was given a placebo in the form of 100 ml of 0.9% NaCl over 20 min; Group B (*n* = 30) received a bolus of MB 1 mg/kg; and Group C (*n* = 30) received a bolus of MB 4 mg/kg in the same manner. Following the first MB bolus dosage in groups B and C, an infusion of 0.25 mg/kg/hour was administered for 72 h. A placebo NaCl infusion syringe was running for 72 h in Group A. Treatment was started within one to two hours of initiating vasopressor administration. All patients received noradrenaline at a rate of 0.1–0.2 µg/kg/min that was adjusted to maintain MAP ≥ 65 mmHg. If noradrenaline at a dose of 0.2 µg/kg/min failed to maintain MAP at 65 mmHg, the patient received hydrocortisone 50 mg/6 hours, which was stopped after 6 h of noradrenaline discontinuation. Infusion bags and intravenous lines were prepared in opaque envelopes in the pharmacy in order not to identify the MB.

Adequate fluid resuscitation is guided by the non-invasive method using an ICON device to assess stroke volume and cardiac output and by inferior vena cava (IVC) collapsibility. The latter is calculated by the difference between the maximum (expiratory) and minimum (inspiratory) IVC diameters, divided by the maximum IVC diameter, and presented as a percentage. This was done 3 times per day as long as the patient was on vasopressors. During the first two hours following MB administration, no adjustments to noradrenaline or sedative/analgesic doses nor ventilator settings were made. After that, noradrenaline was titrated at 20-minute intervals to keep MAP between 65 and 75 mmHg until cessation of infusion.

### Data collection

At randomization, demographic and laboratory data were recorded, and a Sequential Organ Failure Assessment (SOFA) score and a chest X-ray were obtained. Noradrenaline dose and serum lactate levels were documented before starting MB infusion (T0), 2 h (T1), 24 h (T2), 48 h (T3), and 72 h (T4) after the initiation of MB infusion, then after 24 h from MB stoppage (T5). Heart rate (HR), and MAP were measured before and at 30 and 60 min after starting infusion, then every 3 h till the end of infusion. Arterial and mixed venous blood samples were obtained for measurement of PaO_2_, PaCO_2_, O_2_ saturation (SO_2_), and PaO_2_/FiO_2_ before and at 30 and 60 min after initiation of infusion, then every 6 h till the end of infusion. Rapid shallow breathing index (RSBI) is calculated as respiratory rate divided by tidal volume in liters on starting treatment and every 24 h. Creatinine and liver enzymes were also obtained at T0 and every 24 h until cessation of MB infusion and 24 h after.

Toxicity monitoring depended on PaO_2_/FiO_2_ ratio recording to assess oxygenation, a daily ECG to assess cardiac ischemia, and measurement of methemoglobin (MHb) levels. Methemoglobin levels were measured spectrophotometrically in arterial blood as a measure of MB toxicity. Greenish discoloration of skin, mucosa, and urine was clinically observed and was self-limiting.

### Outcome measures

Patients’ follow-up was for 28 days from the day of enrollment. The primary outcome was the time to vasopressor discontinuation. The time was considered valid if discontinuation continued for at least 48 successive hours. Secondary outcomes included total noradrenaline dose, noradrenaline-free days, hemodynamic variables, lactate levels, total duration of mechanical ventilation, length of stay in the ICU and hospital, and changes in creatinine, bilirubin, aspartate/alanine aminotransferase serum levels, PaO_2_/FIO_2_, RSBI, and mortality at 28 days.

### Sample size calculation

The sample size was calculated by G*Power 3.1.9.2 (Universitat Kiel, Germany). A pilot study with 5 cases in each group showed that the mean (± SD) time to vasopressor discontinuation was 92.8 ± 18.4 h in the Control group, 83.2 ± 12.7 h in Low dose group, and 73.8 ± 19.6 h in High dose group. The sample size was based on the following considerations: 0.458 effect size, 95% confidence limit, 95% power of the study, group ratio 1:1:1, and 4 cases were added in every group to overcome dropouts, so 30 patients were in each group.

### Statistical analysis

The statistical analysis was conducted using IBM©, Chicago, IL, USA’s SPSS v27. To assess if the data distribution was normally distributed, the Shapiro-Wilks test and histograms were used. Quantitative parametric data was evaluated by ANOVA (F) test and the post hoc (Tukey) test. Results were provided as mean and standard deviation (SD). Quantitative non-parametric data was compared between each group using the Mann-Whitney test and the Kruskal-Wallis test. Data was given as the median and interquartile range (IQR). The Chi-square test was utilized to examine the frequency and percentage (%) of the qualitative variables. A logistic regression model was used to calculate the hazard ratio of mortality within 28 days considering the control group as the reference. A statistically significant result was defined as a two-tailed P value less than 0.05.

## Results

**Fig. 1:**
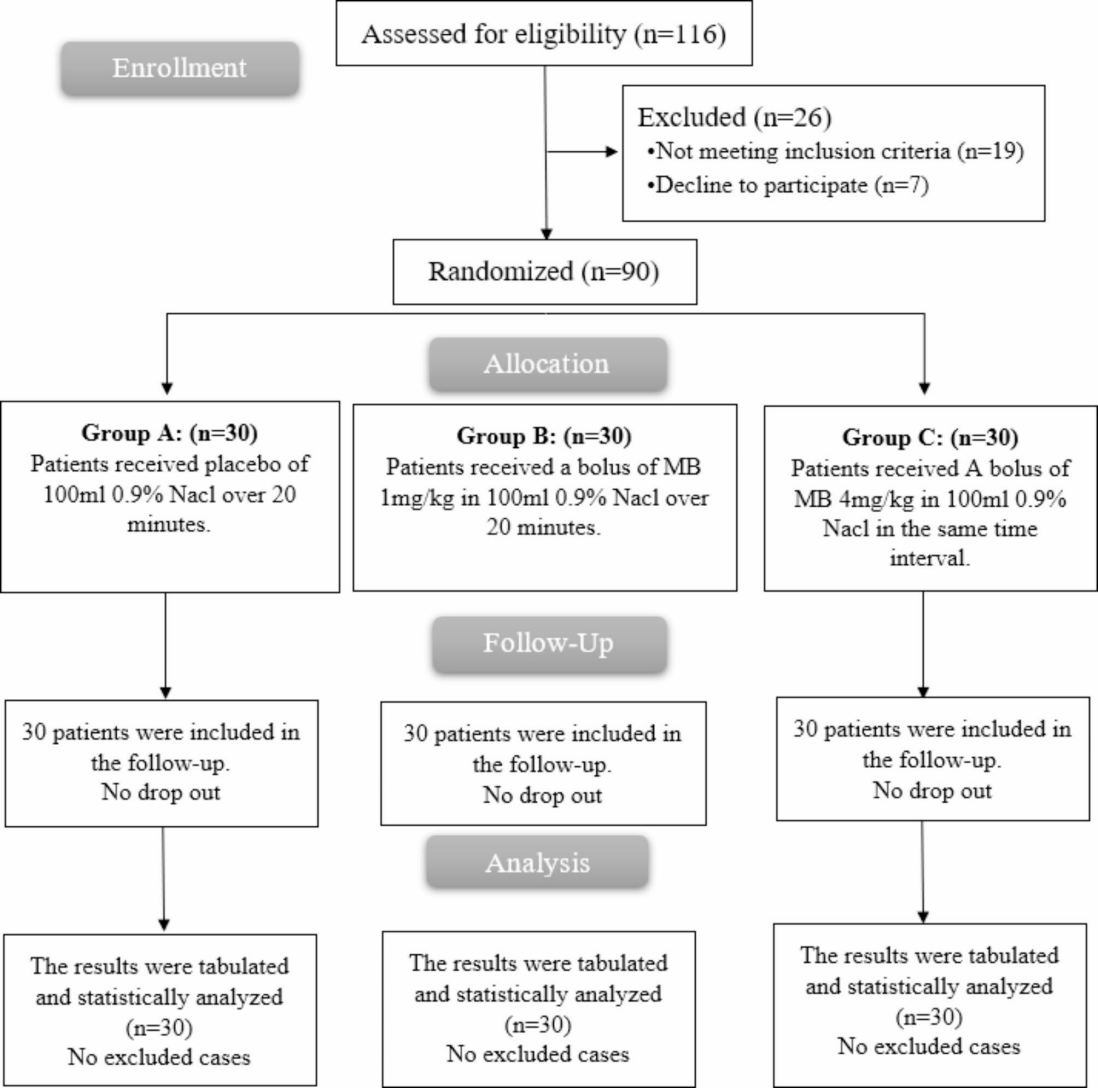
CONSORT flowchart of the included patients

One hundred and sixteen patients were checked for eligibility. Criteria were not met in 19 patients and 7 patients’ guardians refused to be enrolled in the study. Statistical analysis was done for the remaining patients (Fig. 1).

Demographic data, comorbidities, and SOFA scores were insignificantly different among the three groups (Table 1). The patients were suffering breast, pancreatic, and colorectal cancers, with no intergroup differences.

**Table 1:** Demographic data, comorbidities, and SOFA score of the studied groups

		Group A (*n* = 30)	Group B (*n* = 30)	Group C (*n* = 30)	*p*-value
Age (years)		47.8 ± 10.7	46.7 ± 10.3	52.2 ± 11.4	0.125
Sex	Male	20 (66.7%)	18 (60.0%)	22 (73.3%)	0.549
	Female	10 (33.3%)	12 (40.0%)	8 (26.7%)	
Hypertension		11 (36.7%)	10 (33.3%)	8 (26.7%)	0.700
Diabetes mellitus		12 (40.0%)	9 (30.0%)	11 (36.7%)	0.712
Cancer type	Breast	18 (60.0%)	14 (66.7%)	15 (50.0%)	0.768
	Pancreas	3 (10.0%)	6 (20.0%)	6 (20.0%)	
	Colorectal	9 (30.0%)	10 (33.3%)	9 (30.0%)	
Infection source	Pneumonia	16 (53.3%)	14 (46.7%)	15 (50.0%)	0.962
	Intraabdominal	10 (33.3%)	11 (36.7%)	10 (33.3%)	
	Urinary tract	3 (10.0%)	3 (10.0%)	2 (6.7%)	
	Others	1 (3.3%)	2 (6.7%)	3 (10.0%)	
SOFA score		10 (9–12)	10 (9–12)	10 (8–11)	0.185

Data are presented as mean ± SD or frequency (%)

SOFA: Sequential Organ Failure Assessment

**Table 2:** Outcomes of methylene blue treatment of the three studied groups

	Group A (*n* = 30)	Group B (*n* = 30)	Group C (*n* = 30)	*p*-value	Post hoc
Time to vasopressor discontinuation (h)	94.3 ± 17.6	73.6 ± 11.6	70.1 ± 13.4	< 0.001*	p1 < 0.001* p2 < 0.001* p3 = 0.644
Vasopressor-free days at 28 days	23 (0–26)	25 (0–26)	25 (0–26)	< 0.001*	p1 < 0.001* p2 < 0.001* p3 = 0.891
Mechanical ventilation (days)	5 (1–18)	5 (1–13)	4 (1–10)	0.106	
ICU length of stay (days)	12 (3–27)	9 (3–19)	8 (3–17)	0.373	
Hospital stay (days)	27 (3–42)	15 (3–23)	13 (3–22)	0.292	
Mortality rate	14 (46.7%)	9 (30.0%)	6 (20.0%)	0.083	
Hazard Ratio (95% CI)	1 (Ref)	0.49 (0.17–1.41)	0.29 (0.09–0.90)		

Data are presented as mean ± SD, median (range), or frequency (%)

*Significant as p-value < 0.05. p1: Group A vs. Group B, p2: Group A vs. Group C, p3: Group B vs. Group C

Time to vasopressor discontinuation was significantly shorter in Groups B and C compared to Group A. There was no significant difference in the time to vasopressor discontinuation between groups B and C (*p* = 0.644). Likewise, Groups B and C spent significantly more vasopressor-free days than Group A, while there was no significant difference in the vasopressor-free days between Groups B and C (*p* = 0.891). The three groups had comparable mechanical ventilation days, ICU length of stay, and hospital stay. The difference between the three groups in mortality rate was near statistical significance (*p* = 0.083). Using the logistic regression model, the 4 mg/kg group was protective against mortality with a hazard ratio of 0.29 (95%CI: 0.09–0.90) (Table 2).

Noradrenaline doses administered at T0, T1, and T2 time points were comparable between the three groups. The dose at T3 was significantly lower in Group C than in Group A (*p* = 0.026). No significant differences were found at T3 between Groups A and B (*p* = 0.167), or between Groups B and C (*p* = 0.698). Compared to Group A, noradrenaline doses were significantly lower in Groups B and C and not significantly different between Groups B and C at T4 and T5 (Table 2). The total noradrenaline consumption during the 28 days was significantly lower in groups B and C and insignificantly different between groups B and C (Table 3).

**Table 3:** The total noradrenaline dose administered at different time points and the total noradrenaline consumption in the 28 days in the three studied groups

	Group A (*n* = 30)	Group B (*n* = 30)	Group C (*n* = 30)	*p*-value	Post Hoc
Noradrenaline dose (mg)					
T0	0.39 ± 0.12	0.42 ± 0.12	0.46 ± 0.11	0.076	
T1	0.42 ± 0.13	0.41 ± 0.13	0.43 ± 0.12	0.713	
T2	0.42 ± 0.13	0.40 ± 0.12	0.38 ± 0.12	0.533	
T3	0.43 ± 0.13	0.37 ± 0.12	0.34 ± 0.12	0.022*	p1 = 0.167 p2 = 0.026* p3 = 0.698
T4	0.39 ± 0.12	0.30 ± 0.13	0.26 ± 0.12	0.002*	p1 = 0.030* p2 = 0.003* p3 = 0.636
T5	0.31 ± 0.05	0.14 ± 0.06	0.06 ± 0.10	< 0.000*	p1 < 0.001* p2 < 0.001* p3 = 0.284
Total noradrenaline consumption at 28 days (mg)	133.8 (95.7-156.2)	92.9 (83.5–116.0)	82.9 (63.1–116.0)	< 0.001*	p1 = 0.002* p2 < 0.001* p3 = 0.751

Data are presented as mean ± SD or median (IQR)

*Significant as p-value < 0.05. p1: Group A vs. Group B, p2: Group A vs. Group C, p3: Group B vs. Group C

Serum lactate was insignificantly different at T0, T1, T2, and T3 among the three groups, was significantly lower in Groups B and Group C compared to Group A, and was insignificantly different between Group B and Group C at T4 and T5.

**Fig. 2:**
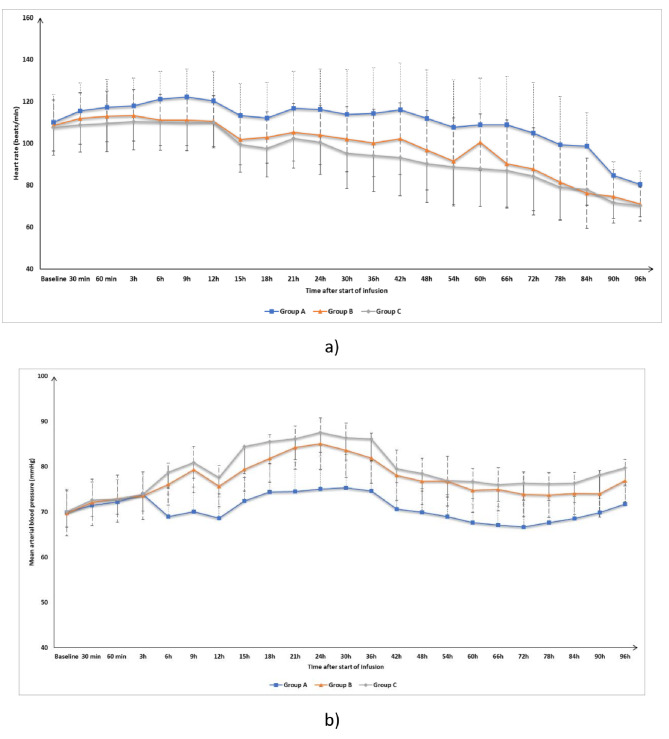
(**a**) Heart rate, (**b**) Mean arterial blood pressure of the three groups

Heart rate and MAP were insignificantly different at T0, 30 min, 60 min, and 3 h after the start of infusion among the three groups. The heart rate was significantly lower in Groups B and C compared to Group A and was insignificantly different between Group B and Group C at all time points from 6 to 96 h after the start of infusion (Fig. 2a). Mean arterial blood pressure was significantly higher in Groups B and C compared to Group A and was insignificantly different between Group B and Group C at all time points from 6 to 96 h after the start of infusion (Fig. 2b).

**Fig. 3:**
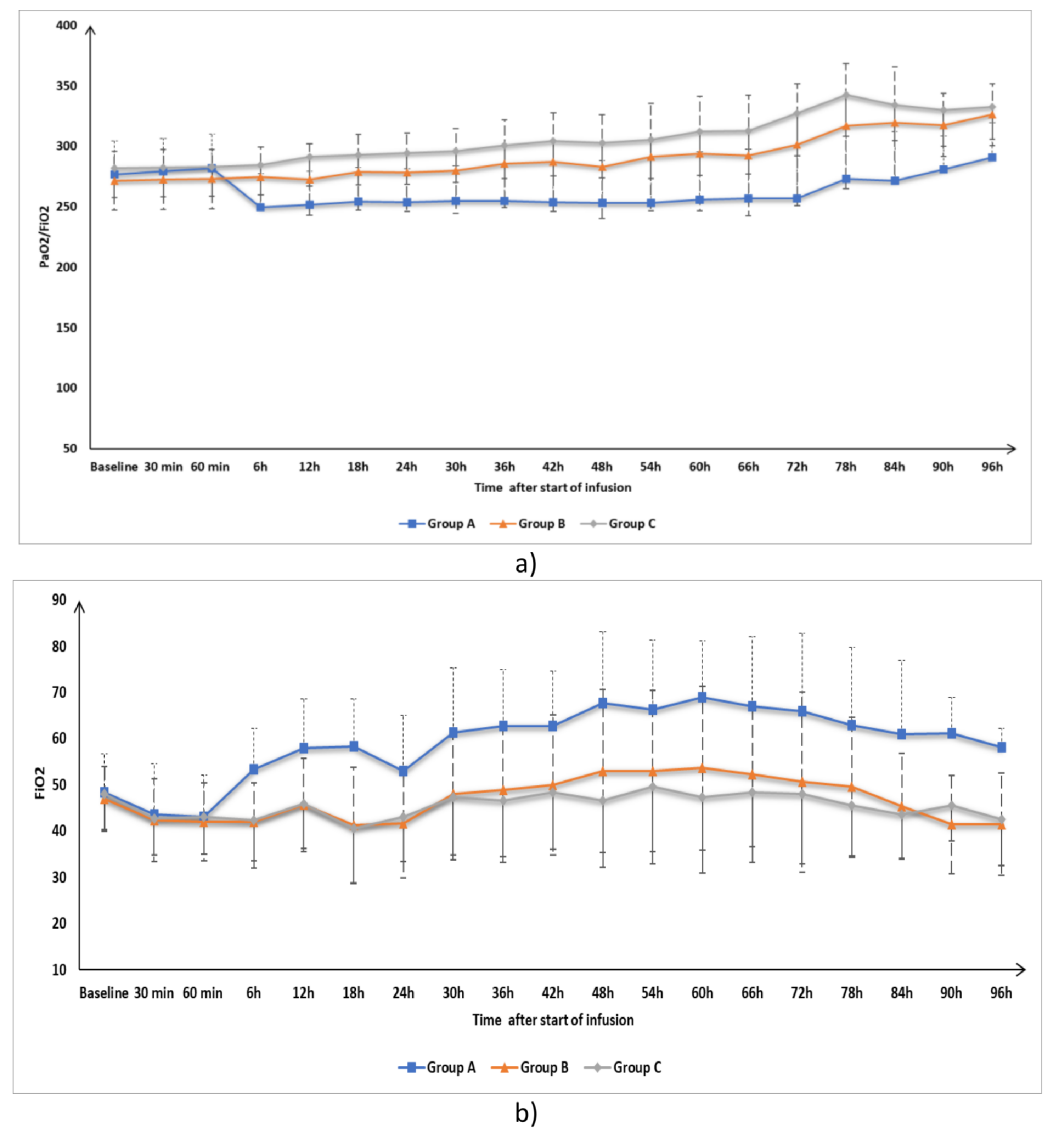
(**a**) PaO_2_/FiO_2_ (**b**) FiO_2_ of the three groups

FiO_2_ and PaO_2_/FiO_2_ were insignificantly different at T0, 30 min, 60 min, and 3 h after the start of infusion among the three groups. PaO_2_/FiO_2_ was significantly higher in Groups B and C compared to Group A (Fig. 3a), while FiO_2_ was significantly lower in Groups B and C compared to Group A (Fig. 3b). PaO_2_/FiO_2_ and FiO_2_ were insignificantly different between Group B and Group C at all time points from 6 to 96 h after the start of infusion (Fig. 3a and b).

RSBI was insignificantly different at T0, T1, T2, T3, and T4 among the three groups. RSBI was significantly lower in Groups B and C compared to Group A (*p* < 0.05) and was insignificantly different between Groups B and C at T5. ALT, AST, and creatinine were insignificantly different at T0, T1, T2, T3, T4, and T5 among the three groups.

A daily ECG done for all patients revealed some ECG ischemic changes in a few patients; two in group A (0.6%), three in group B (0.9%), and five in group C (1.5%). The results were statistically insignificant. These changes were transient and disappeared while the MB infusion was continuous. There was no effect on MHb levels up to 24 h after cessation of MB infusion.

## Discussion

Several clinical trials and meta-analyses provided evidence indicating that MB may decrease short-term mortality in patients with septic shock. Additional findings indicate that the injection of MB may improve hemodynamic status, elevate MAP, decrease the duration of vasopressor exposure, and shorten the time of hospitalization. However. different dose regimens of MB were evaluated varying from bolus delivery to bolus administration followed by continuous infusion, or continuous infusion only. So far, no research has explored the ideal dosage strategy for patients with septic shock. Based on a recent study [10], we adopted the bolus + continuous infusion strategy. This strategy was found to be associated with decreased 28-day mortality. We used a 4 mg/kg bolus dose aiming to enhance the effectiveness of MB based on a previous dose-finding study [11]. This was a prospective, randomized, double-blind study that tested 3 bolus doses of MB, 1 mg/kg, 3 mg/kg, and 7 mg/kg over 20 min in mechanically ventilated patients with septic shock. The authors concluded that MB increases MAP, improves left ventricular filling and function, and raises cardiac output in a *dose-dependent manner*. We hypothesized that the high dose would be more effective in the current group of cancer patients with septic shock. Nevertheless, our results did not completely substantiate this idea.

In this prospective study, the 1 mg/kg and 4 mg/kg bolus doses were almost equally effective adjuvants for the treatment of septic shock. We found that MB had a positive impact on noradrenaline requirement in septic shock as it significantly reduced the time to discontinue vasopressor administration and vasopressor-free days compared to the placebo group. Noradrenaline doses over time points and its total consumption at 28 days were significantly lower with the use of MB irrespective of the bolus dose used. Serum lactate was lower and MAP higher in the methylene blue groups. PaO_2_/FiO_2_ and RSBI also improved with the use of methylene blue. However, the effect on mortality of the two used doses was found to be insignificant. Nevertheless, an important observation was that the 4 mg/kg dose had a survival advantage when compared to the control group with a hazard ratio of 0.29 (95%CI: 0.09–0.90). We do not have a clear explanation of this survival advantage of the high-dose bolus. However, this might stem from early intensified control of noradrenaline action, which may limit its serious adverse effects.

Similar outcomes of MB use were reported in previous trials with different dosing regimens. A bolus of 4 mg/kg of MB over 1 h was investigated in ten patients with severe septic shock. The authors showed a positive impact on systemic parameters including arterial pressure, systemic vascular resistance (SVR), and left ventricular stroke volume. However, it exerted a negative impact on pulmonary circulation and gas exchange [12]. Although they showed a reduction of serum lactate [13], they could not explain it by improvement in tissue perfusion. This may be due to an improvement in oxygen utilization at a cellular level or a direct reduction property of MB [14]. Worth noting is that in this pilot study patients were already suffering from severe septic shock and respiratory failure. With the same high bolus dose of 4 mg/kg, we did not observe a negative adverse effect on oxygenation or gas exchange. In the current study, serum lactate showed a continuous reduction that extended to 24 h after cessation of MB infusion.

In refractory septic shock, 1 mg/kg MB infusion increased SVR and MAP [15]. A bolus of 2 mg/kg MB, followed by infusion at an increasing rate of 0.25, 0.5, 1, and 2 mg/kg/hr for 1 h lowered the noradrenaline requirement in septic shock patients [16]. MB infusion at a dose of 0.5 mg/kg/hr for 6 h positively impacted MAP with no effect on mortality rates [17]. A more recent randomized trial examined the efficacy of a daily continuous infusion of 100 mg of MB over 6 h for 3 days in septic shock. MB infusion was associated with a shorter time to vasopressor discontinuation, more vasopressor-free days, and a shorter length of stay in the ICU and hospital compared to a placebo control group. No mortality benefit was noted [18]. A meta-analysis of 6 RCTs including 214 patients showed that MB improved MAP and decreased serum lactate with a non-significant decrease in mortality [19].

A case study showed that a bolus of 3 mg/kg for 20 min, followed by 0.5 mg/kg/h for 48 h early in septic shock reduced lactate levels, inflammatory cytokines, and the dose of vasopressors. Twenty-four hours after the MB was suspended, there was a rise in serum lactate and a return of noradrenaline to 0.03 µg/kg/min [20]. This finding may be an indicator of the importance of continuous MB infusion to help maintain hemodynamics and tissue perfusion.

We believe that MB as a bolus + continuous infusion might prolong suppression of the NO pathway, which is accomplished in two phases: iNO synthase inhibition [21] and the downstream sGC [22]. Nevertheless, we failed to prove the hypothesis of a higher effect with a high bolus dose of MB. This may be attributed to the unpredictable duration of systemic inflammation in septic shock [23]. Thus, the cumulative dose of MB appears more valuable than the bolus dose in septic shock, probably due to a half-life of 5–6 h [19].

Elevated MB in whole blood exceeds plasma concentration, indicating accumulation in blood cells. Acute inflammation triggers NO generation by immune cells, including macrophages, therefore augmenting the inflammatory stimulus. This might suppress an excessive inflammatory reaction in septic shock, which leads to hemodynamic derangement [24].

MB has the advantage of being a selective inhibitor of iNOS, sparing the constitutive isoform, therefore enhancing microcirculatory flow, increasing blood flow to ischemic regions, scavenging oxygen-free radicals, and promoting microbial death by macrophages [25]. Also, MB can act as an antioxidant to decrease oxidative stress and lower plasma levels of IL-6 [26].

When using MB in septic shock treatment, several points should be considered. One is toxicity. A one-time bolus of 1–2 mg/kg MB was shown to be safe in sepsis and was not associated with the development of methemoglobinemia [27]. The current study provided evidence of the safety of a 4 mg/kg bolus dose. We did not observe adverse effects of MB on the circulatory, respiratory, or metabolic levels. Besides, no cases of methemoglobinemia were recorded. The continuous and extended infusion of MB can result in increased cumulative dosages, which may have hazardous effects [11]. A recommended total cumulative dose of 5.75 mg/kg for continuous infusion in cases of septic shock was recommended [28]. Higher dosages may result in methemoglobinemia. Therefore, the current work made use of a dosage of 0.25 mg/kg/h.

Another point is cardiac ischemia. In this study, some ECG ischemic changes in a few patients were identified. These changes were transient and disappeared while the MB infusion was continuous. It may be attributed to a temporary loss of the beneficial vasodilatory effects of NO on myocardial blood flow due to MB administration [29].

### Limitations

Some limitations should be admitted. First, this was a single-center study. However, as the largest reference center for cancer care in Egypt, this study exhibited the results of using MB in a special group of patients with different types of cancer as the primary disease. They were relatively younger than patients admitted to the ICUs of general hospitals with septic shock. Most patients were already managed in other departments of the NCI while diagnosed with septic shock. Therefore, nosocomial infection is a very common occurrence in such a patient group. Another limitation is that we did not study the other factors contributing to mortality to reach a robust conclusion about the effect of MB on mortality. We did not measure cytokine or nitrate/nitrite serum levels to settle the mechanism of the effects of MB. The study lacked complete blinding due to the presence of bluish coloration on the skin and secretions caused by the administration of methylene blue. However, we kept the blinding of patients and outcome assessors for groups allocated to low and high MB doses.

While acknowledging these limitations, our work contributes to the existing knowledge on the impact of MB on septic shock. To our knowledge, the present work is the first to provide a comparison between the standard bolus dosage of 1 mg and a high dose of 4 mg/kg. Hence, we have determined that MB is safe at this targeted dosage in cancer patients.

*In conclusion*, in septic shock, early adjunctive MB delivery during the first two hours of vasopressor initiation was associated with early discontinuation and reduced total consumption of noradrenaline. The 1 mg/kg and 4 mg/kg bolus doses of MB were similarly effective. Compared to the placebo control group, the 4 mg/kg bolus dose had a survival advantage. However, there was no significant effect on mortality for the 1 mg/kg bolus. No major side effects were observed. There were no significant adverse effects in both dose regimens.

We recommend initiating MB treatment in a bolus dose of 4 mg/kg bolus dose followed by 0.25 mg/kg/hour for 72 h in patients with septic shock within 2 h of starting vasopressors. Monitoring of possible adverse effects is essential during MB treatment.

## Data Availability

The datasets generated during and/or analyzed during the current study are available from the corresponding author on reasonable request.

## Abbreviations

MB: Methylene blue
QSOFA: The Quick Sequential [Sepsis-related] Organ Failure Assessment
MAP: A mean arterial pressure
